# Assessment of the mechanism of drug resistance in *Trichophyton mentagrophytes* in response to various substances

**DOI:** 10.1186/s12864-021-07520-6

**Published:** 2021-04-07

**Authors:** Chenwen Xiao, Jiaoyu Wang, Zhenfeng Liao, Yee Huang, Quanan Ji, Yan Liu, Fei Su, Lijun Xu, Qiang Wei, Yao Pan, Ke Li, Guolian Bao

**Affiliations:** 1 Institute of Animal Husbandry and Veterinary Science, Zhejiang Academy of Agricultural Sciences, Hangzhou, China; 2 State Key Laboratory for Managing Biotic and Chemical Treats to the Quality and Safety of Agro-products, Institute of Plant Protection and Microbiology, Zhejiang Academy of Agricultural Sciences, Hangzhou, 310021 China; 3 Central Laboratory of Zhejiang Academy of Agricultural Sciences, Zhejiang Academy of Agricultural Sciences, Hangzhou, China; 4 National Clinical Research Center for Infectious Diseases, The Department of Infectious diseases, State Key Laboratory for Diagnosis and Treatment of Infectious Diseases, The First Affiliated Hospital, College of Medicine, Zhejiang University, 79 Qingchun Road, Hangzhou, 310003 China; 5 College of Life Sciences, China Metrology University, Hangzhou, China

**Keywords:** Trichophyton mentagrophyte, Steroid pathway, ERG25, CYP450, Transcriptome, Proteomics

## Abstract

**Background:**

Trichophyton mentagrophyte (TM), a zoonotic pathogen, has been endangering public health due to emerging drug resistance. Although increased attention is paid to this issue, there is very limited research available on drug resistance in TM. In this study, we studied the gene and proteomic changes, morphological changes, cellular fat localization, fat content changes, and biofilm of TM treated with different substances.

**Results:**

The TM growth curve showed a positive correlation with the concentration of Fenarimol (FE), genistein (GE), clotrimazole (KM), and Miconazole nitrate salt (MK). The morphology of TM cells changed in different degrees after treatment with different substances as observed by TEM and SEM. The results showed that under KM and berberine hydrochloride (BB) treatment, a total of 3305 differentially expressed genes were detected, with the highest number in the KM-treated group (578 up-regulated and 615 down-regulated). A total of 847 proteins and 1850 peptides were identified in TM proteomics. Nile red staining showed that the fat content of TM was significantly higher in the BB-, ethidium bromide- (EB), FE-, KM-, Adriamycin hydrochloride- (YA), and MK-treated group compared to the control group. Results of the biofilm thickness showed that it gradually increased under treatment with specific concentrations of KM or BB, which may be related to the up-regulation of ERG25 and CYP related gene proteins.

**Conclusions:**

It is suggested that in order to effectively deal with dermatomycosis caused by TM, it is necessary to inhibit the expression of ERG25 and CYP related genes and fat metabolism, which can result in the inhibition of the production of biofilm by the fungus and solve the problem of fungal drug resistance in clinical settings.

**Supplementary Information:**

The online version contains supplementary material available at 10.1186/s12864-021-07520-6.

## Background

Trichophyton mentagrophyte (TM), a zoonotic pathogen, has been endangering public safety as dermatomycosis caused by TM is one of the most common skin diseases in the rabbit industry. It mainly damages the fur and affects its quality. In serious cases, it can lead to malnutrition, growth retardation, and reduced feed returns, which causes great economic losses to the rabbit industry [1]. Although there are a variety of clinical drugs for the treatment of animal fungal skin diseases, including hormones, antibiotics, and antifungal drugs, they have many disadvantages, such as drug resistance with long-term use side effects [2]. At present, the mechanism of drug resistance in TM is still unclear and only a few studies on functional genes have been published [3–5]. In this study, we used different substances to evaluate the potential mechanism of drug resistance in TM, including ethidium bromide (EB), genistein (GE), Congo red (GR), Dimethyl sulfoxide (DMSO), clotrimazole (KM), berberine hydrochloride (BB), Miconazole nitrate salt (MK), Fenarimol (FE), Adriamycin hydrochloride (YA), and Sabouraud dextrose agar (SDA) as reference control. The growth curve, (scanning) electron microscope observation, transcriptome sequencing analysis, and fluorescent quantitative PCR verification were carried out to evaluate them. Proteomics analysis of some substances was also performed. To further analyze the mechanism of drug resistance in TM exposed to these substances, the content of ergosterol was analyzed and the Nile red staining and biofilm thickness were observed through a confocal microscope. The results lay a foundation for scientific prevention and treatment of dermatomycosis caused by TM.

## Results

### Growth curve of TM after treatment with different substances

**Fig. 1:**
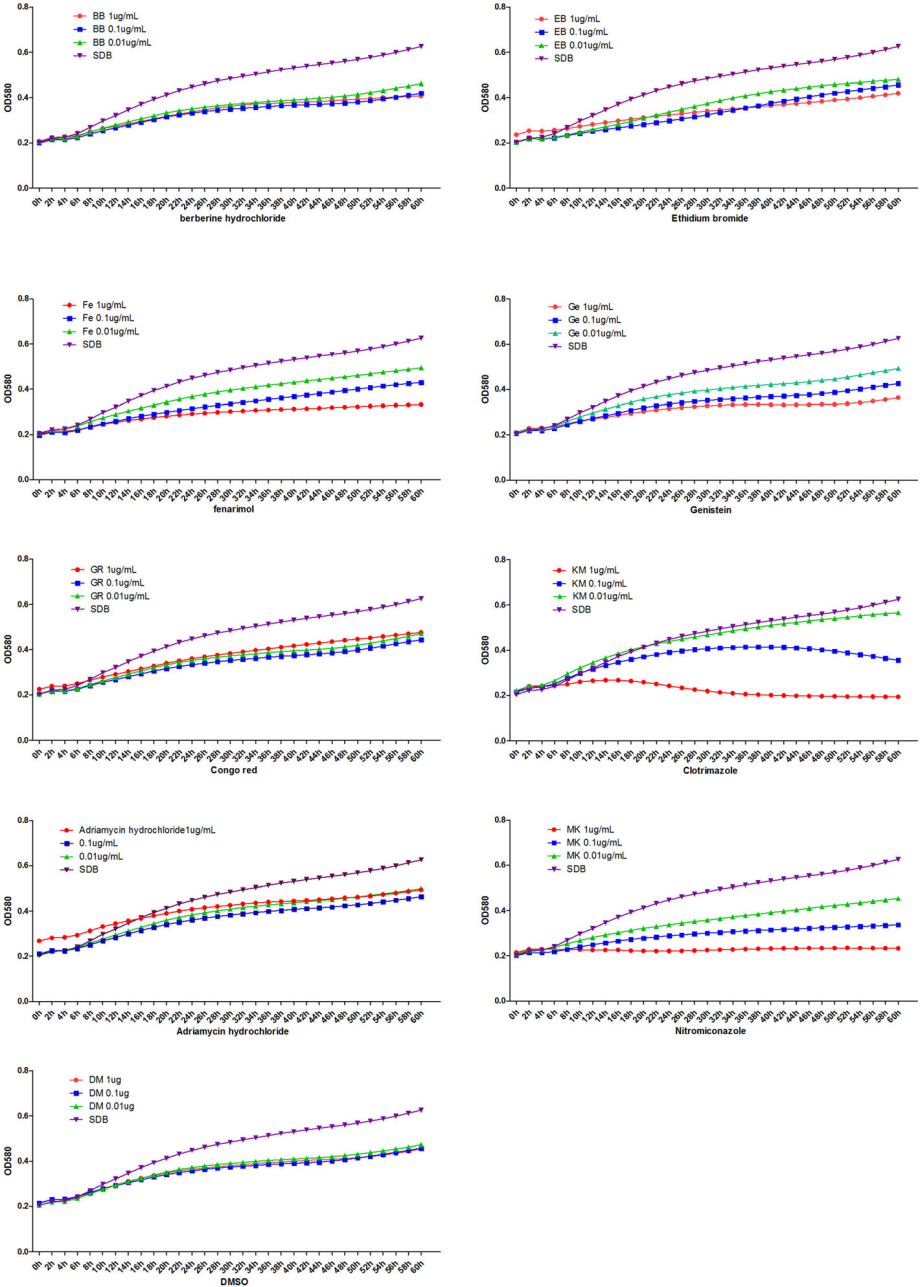
The effects of varying concentrations of different test substances on TM were determined over time as described in the methods section. Sabouraud dextrose agar (SDA) medium served as a reference control

Figure 1 shows the growth curve of TM after treatment with different substances. The growth curves of the BB- and YA- treated group in three different concentrations showed little difference. The growth of TM was slightly stronger at a low concentration of both EB and GR, compared to that of medium and high concentrations, although the differences were minor in GR. The trend of TM’s growth curve showed a positive correlation with the concentration of FE, GE, KM, and MK. DM seemed to have a minor effect on the growth of TM.

### The morphological changes of TM

**Fig. 2:**
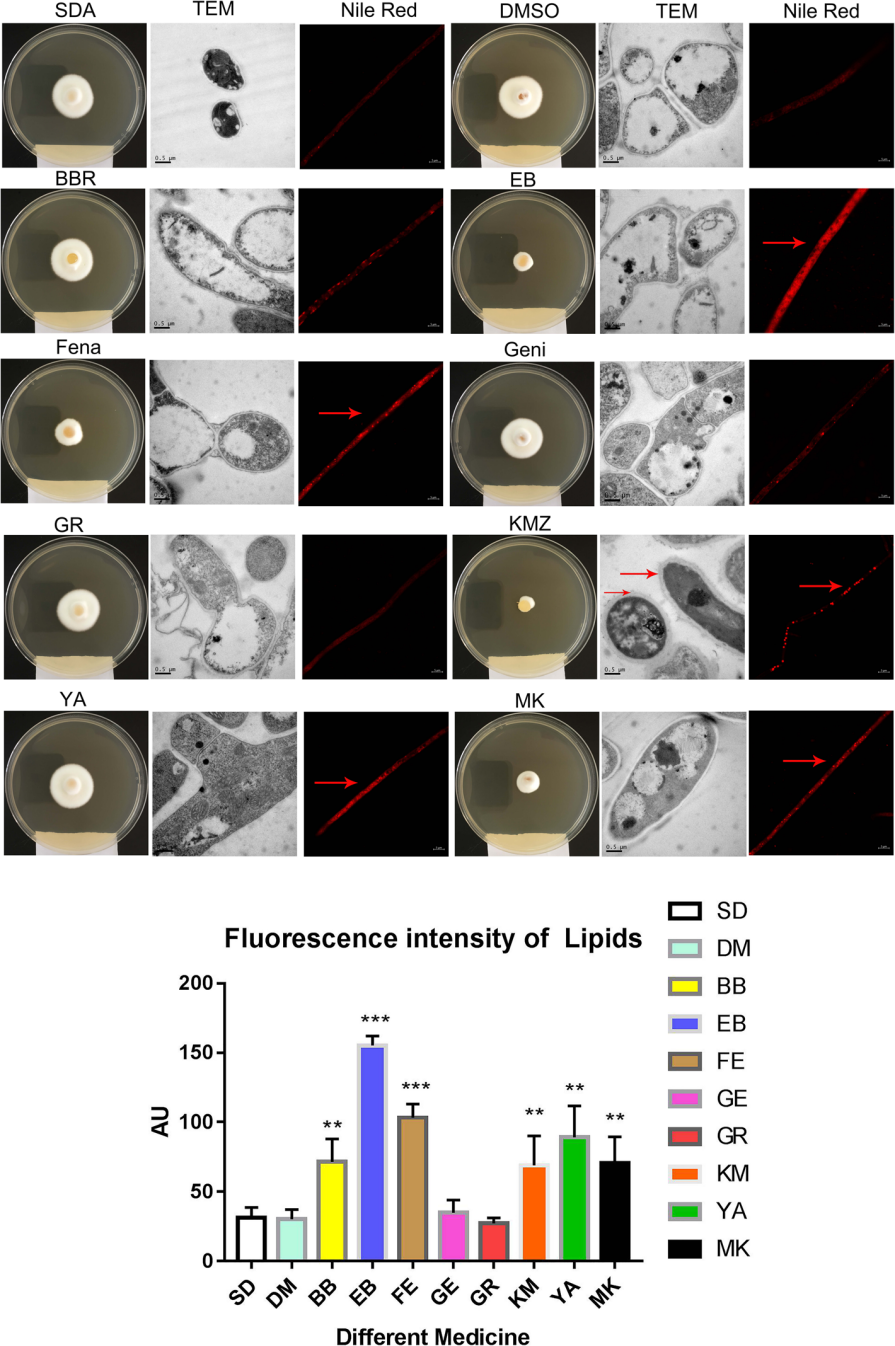
Observation of morphological changes of TM cells following the addition of test substances under a Zeiss confocal microscope) and, Nile red staininged with Nile red. and observed under a Zeiss confocal microscope. The following test substances were used: Sabouraud dextrose agar (SDA), Dimethyl sulfoxide (DMSO), berberine hydrochloride (BB), ethidium bromide (EB), Fenarimol (FE), genistein (GE), Congo red (GR), clotrimazole (KM), Adriamycin hydrochloride (YA), and Miconazole nitrate salt (MK)

Observation through a TEM showed, that the morphology of TM cells had different degrees of changes following treatment with various substances (Fig. 2). In the SDA-treated group, the morphology of TM was normal. After DMSO treatment, the morphology of TM changed slightly, which was mainly reflected by the appearance of granular substance in its cytoplasm. After BB treatment, TM’s membrane became folded and damaged. While in the EB-treated group, the cell membrane of TM became damaged, the nucleus stained intensively, the mitochondria could be clearly identified, and the red color was bright and diffuse after Nile red staining. After FE treatment, TM’s cell membrane became damaged, granular-like substances appeared, and the staining turned bright red with red particles. TM treated with GE also had damaged cell membranes. TM cells seemed bulky, granular material appeared, and the cell membrane was damaged following GR treatment. After KM treatment, TM’s cell membrane was obviously thickened, the nucleus condensed, and granular substance appeared as well. In addition, in the YA-treated group, the cell membrane became indistinct, mitochondria clearly visible, and the red color was bright and diffuse after staining. Following MK treatment, TM’s cell membranes became damaged, the nucleus condensed, the endoplasmic reticulum clearly visible, and after staining, the red color was bright and distributed as fine particles. The fluorescence intensity of the Nile red staining was consistent with confocal microscopy observations; the fat content of TM in the BB-, EB-, FE-, KM-, YA-, and MK-treated groups were significantly higher compared to that of the control group, implying that the results of the Nile red staining were reliable.

### SEM observation of morphological changes of TM

**Fig. 3:**
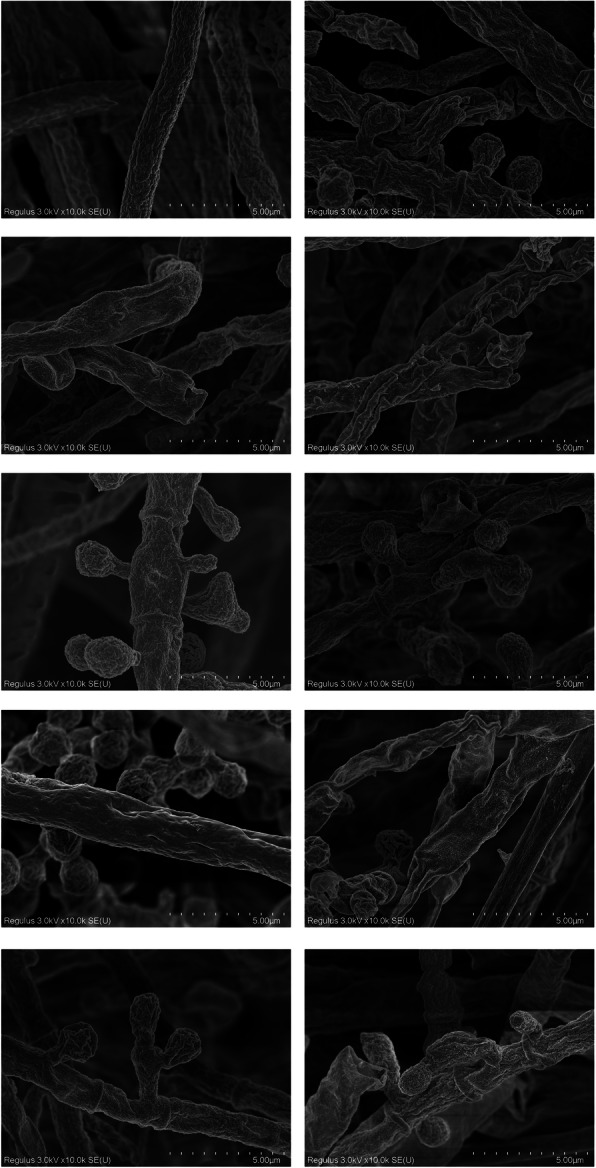
SEM analysis of the TM specimens following exposure to different drugssubstances. The specimens were placed in ethanol:iso-amyl acetate (1:1) for 30 min, and then in iso-amyl acetate overnight

It was observed through SEM that the morphology of TM cells in the groups treated with various substances was different from that of the control group (Fig. 3). In the DMSO-treated group treatment, the morphology of TM changed slightly, which was mainly reflected by shrinking of its shape. TM cells shrank and some of them broke in the BB-treated group. While in the EB-treated group, TM cells were shrunken even more and the hyphae were damaged. In the FE-treated group, the spores of TM cells were deformed or reduced. In the GE-treated group, TM cells were deformed and some spores were broken. The surface of TM cells became smooth in the GR-treated group. In the KM-treated group, the spores of TM became deformed, the mycelium shrunken, and the surface smooth. In the YA-treated group, the spores became smaller and the mycelium shrank. And lastly, TM’s cell membrane broke and the spores became deformed in the MK-treated group.

### TM transcriptome changes and gene ontology classification results

**Fig. 4:**
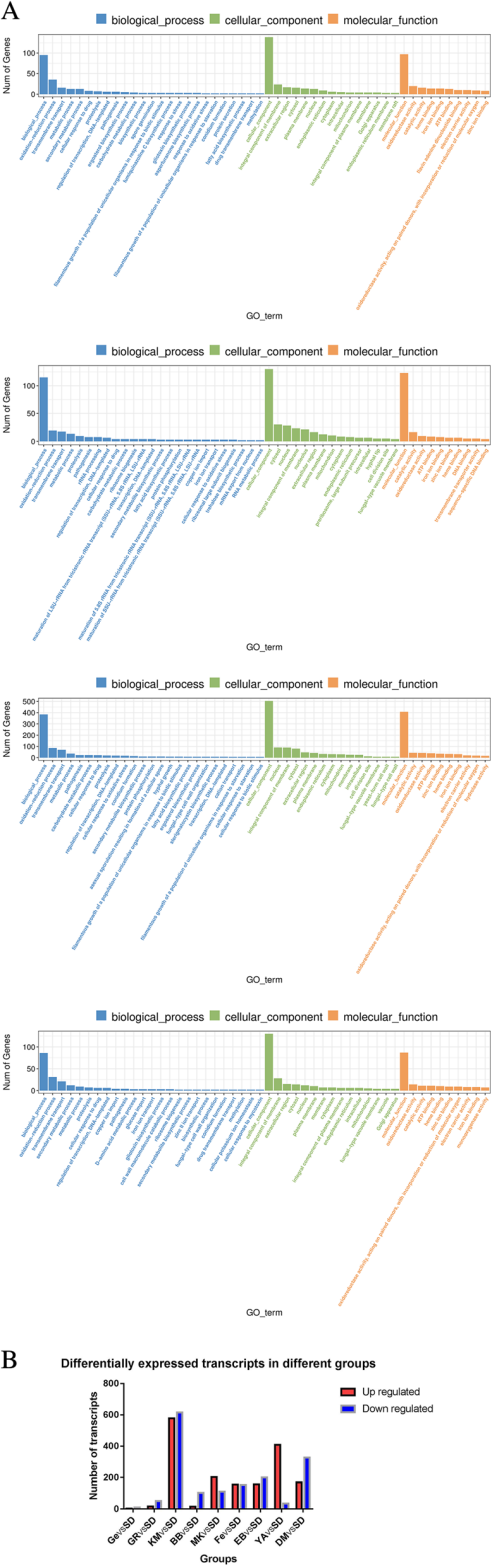
**a**: GO classification of TM after exposure to FE, EB, KM and MK in comparison to the control group. **b**: Differentially expressed genes (DEGs) of the different groups, of which the KM-treated group consisted of the most up- and down-regulated DEGs. All the results were statistically significant (*P* < 0.05)

According to the Gene Ontology (GO) classification as shown in Fig. 4a, the most enriched GO’s in the FE-treated group were biological process, oxidation process, cellular component, integral component of membrane, molecular function, and oxidoreductase activity. The most enriched GO’s in the EB-treated group were biological process, oxidation-reduction process, transmembrane transport, cellular component, cytosol, molecular function, and catalytic activity. The most enriched GO’s in the KM-treated group were biological process, oxidation-reduction process, transmembrane transport, cellular component, nucleus, molecular function, and catalytic activity. The most enriched GO’s in the MK-treated group were biological process, oxidation-reduction process, transmembrane transport, cellular component, integral component of membrane, molecular function, and oxidoreductase activity. These data have been deposited in the Sequence Read Archive of the National Center for Biotechnology Information (NCBI) (accession number, GSE162331; [NCBI tracking system #21491828]). Figure 4b shows the differentially expressed genes (DEGs) of the different groups (*P* < 0.05). In total 3305 DEGs were detected. Among them, 3 were up-regulated and 9 down-regulated (*P* < 0.05) in the GE-treated group. In the GR-treated group, 16 were up-regulated and 51 down-regulated (*P* < 0.05). The KM-treated group had 578 up-regulated and 615 down-regulated genes (*P* < 0.05). The BB-treated group consisted of 15 up-regulated and 102 down-regulated genes (*P* < 0.05). In the MK-treated group were 202 up-regulated and 110 down-regulated genes (*P* < 0.05). The FE-treated group had 156 up-regulated and 153 down-regulated genes (*P* < 0.05). The EB-treated group had 157 up-regulated and 200 down-regulated genes (P < 0.05). The YA-treated group had 408 up-regulated and 34 down-regulated genes (P < 0.05). And lastly, the DMSO-treated group had 169 up-regulated and 327 down-regulated DEGs (P < 0.05).

### RT-PCR verification of transcriptome

**Fig. 5:**
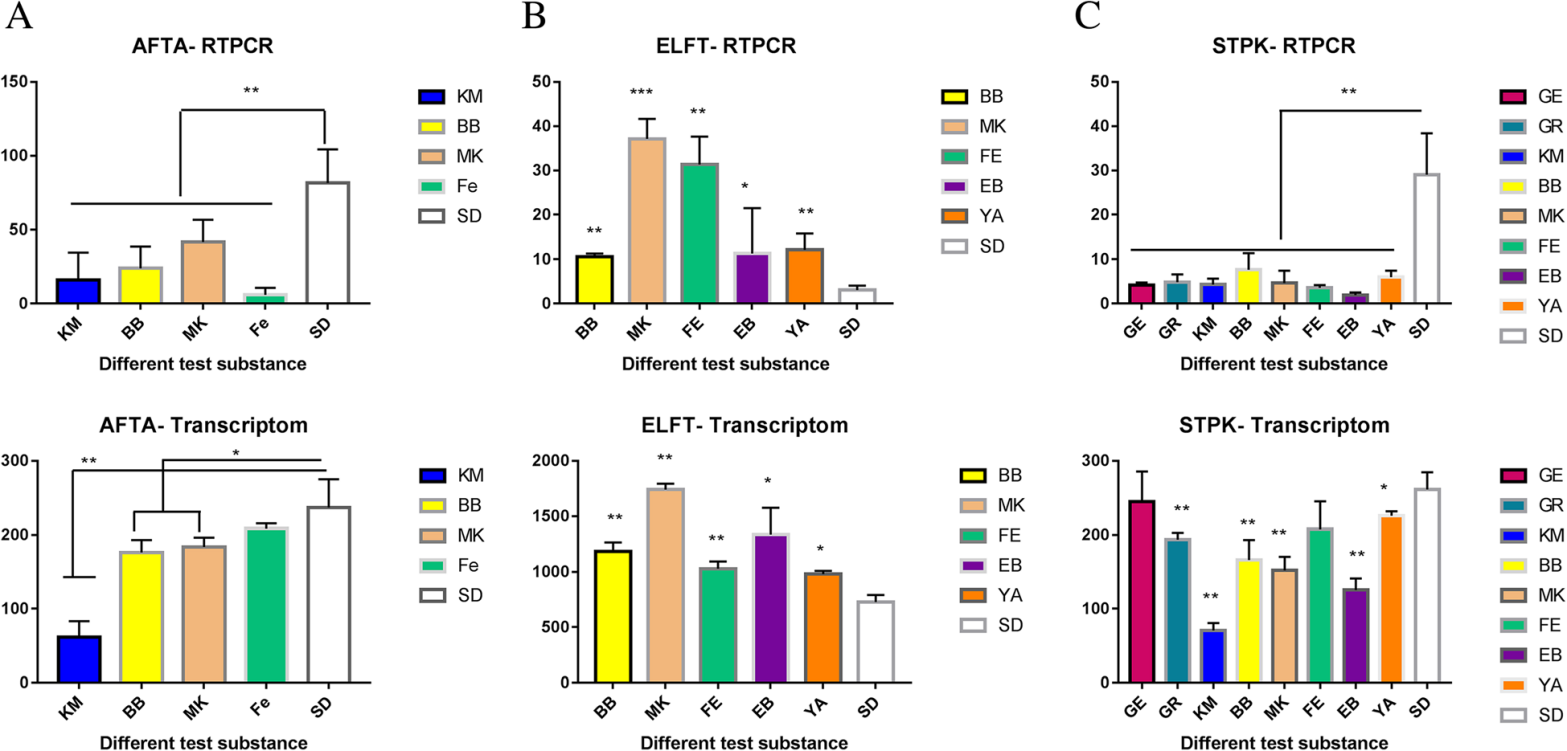
**a** shows the results of RT-PCR and transcriptome of the AFTA gene. **b** shows the results of RT-PCR and transcriptome of the ELFT gene. **c** shows the results of RT-PCR and transcriptome of the STPK gene. There were significant differences between the groups exposed to the test substances and SDA control group, *P < 0.05, **, *P* < 0.01, ***, *P* < 0.001

Comparison of the results of RT-PCR and transcriptome showed that several treatments of AFTA and ELFT genes were completely consistent (Fig. 5). The RT-PCR results of the STPK gene in the GE- and FE-treated group indicated a significant lower fold change compared to the SDA (control) group, although no significant differences were observed between these groups in the transcriptome results (Fig. 5). These results indicate that the transcriptome results were reliable.

### Changes in TM proteomics after KM and BB treatment

**Fig. 6:**
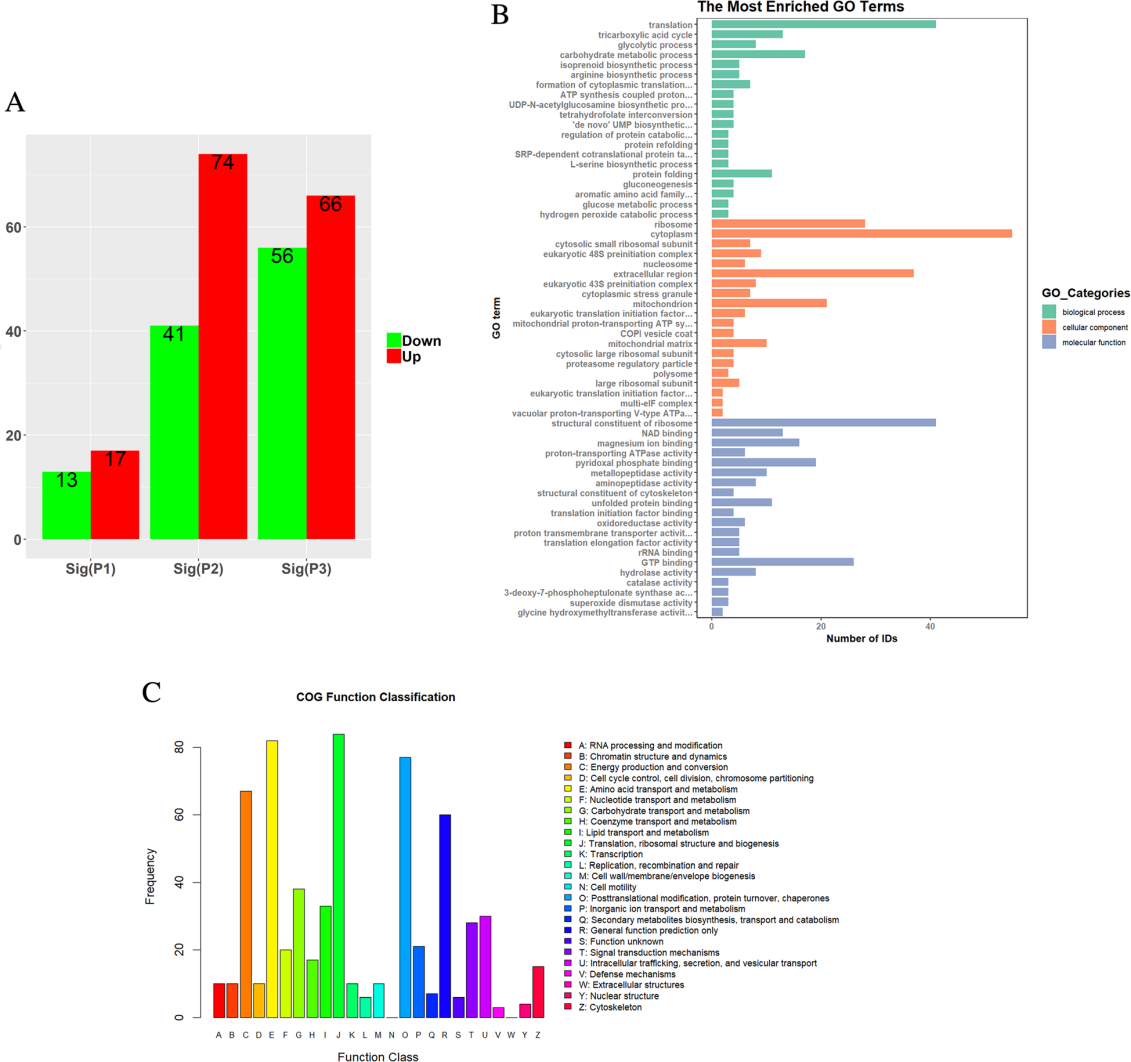
**a**: Results of TM proteomics under KM and BB treatments. The selection criteria were *P* ≤ 0.05 and fold change of 1.5 times the significant difference. P1 represents BB vs SDA, P2 represents KM vs SDA, and P3 represents KM vs BB. **b**: DAPs were described by the most enriched GO terms and also categorized into the three following GO categories: “cellular component,” “biological process,” and “molecular function”. **c**: COG analysis is based on the homologous classification of gene products in the COG database. The most frequently detected COG were: “Translation, ribosomal structure and biogenesis”, “Posttranslational modification, protein turnover, chaperones”, “Amino acid transport and metabolism”, and “Energy production and conversion ”

We identified 847 proteins and 1850 peptides in the KM- and BB-treated groups with proteomics. In the BB-treated group, 17 proteins were up-regulated and 13 proteins down-regulated. In the KM-treated group, 74 proteins were up-regulated and 41 proteins down-regulated. In comparison with the BB-treated group, there were 66 up-regulated proteins and 56 down-regulated proteins in the KM-treated group. The selection criteria were *P* ≤ 0.05 and fold change 1.5 times the significant difference (Fig. 6a). The following GO terms were used to describe DAPs: “translation,” “ribosome,” “structural constituent of ribosomal subunit,” and “extracellular region” (Fig. 6b). In addition, the DAPs were also classified according to the following three GO categories: “biological process”, “cellular component”, and “molecular function” (Fig. 6b). COG analysis is based on homologous classification of gene products in the COG database. Our results showed that the most frequently found COG were: “Energy production and conversion”, “Amino acid transport and metabolism”, “Translation, ribosomal structure and biogenesis”, and “Post translational modification, protein turnover, chaperones” (Fig. 6c).

### Functional annotation of differentially expressed genes such as KEGG and GO enrichment

Gene name of methylsterol monooxygenase with Gene ID TMEN_7126 showed significant expression in certain substances treatment groups such as EB-, Fe-, KM-, MK-. The KEGG pathway of TMEN_7126 is belonged with 00100 (Steroid biosynthesis). And Go annotation is GO:0000254(C-4 methylsterol oxidase activity);GO:0005506(iron ion binding);GO:0005789(endoplasmic reticulum).

Another Gene name of cytochrome P450 with Gene ID TMEN_2906 showed significant expression in certain substances treatment groups such as Fe-, KM-. The KEGG pathway of TMEN_2906 is belonged with 00404(Staurosporine biosynthesis). And Go annotation is GO:0003824(catalytic activity);GO:0005506(iron ion binding);GO:0005575(cellular_component);GO:0008202(steroid metabolic process);GO:0009055(electron carrier activity);GO:0016705(oxidoreductase activity, acting on paired donors, with incorporation or reduction of molecular oxygen);GO:0020037(heme binding);GO:0055114(oxidation-reduction process).

### Determination of ergosterol

**Fig. 7:**
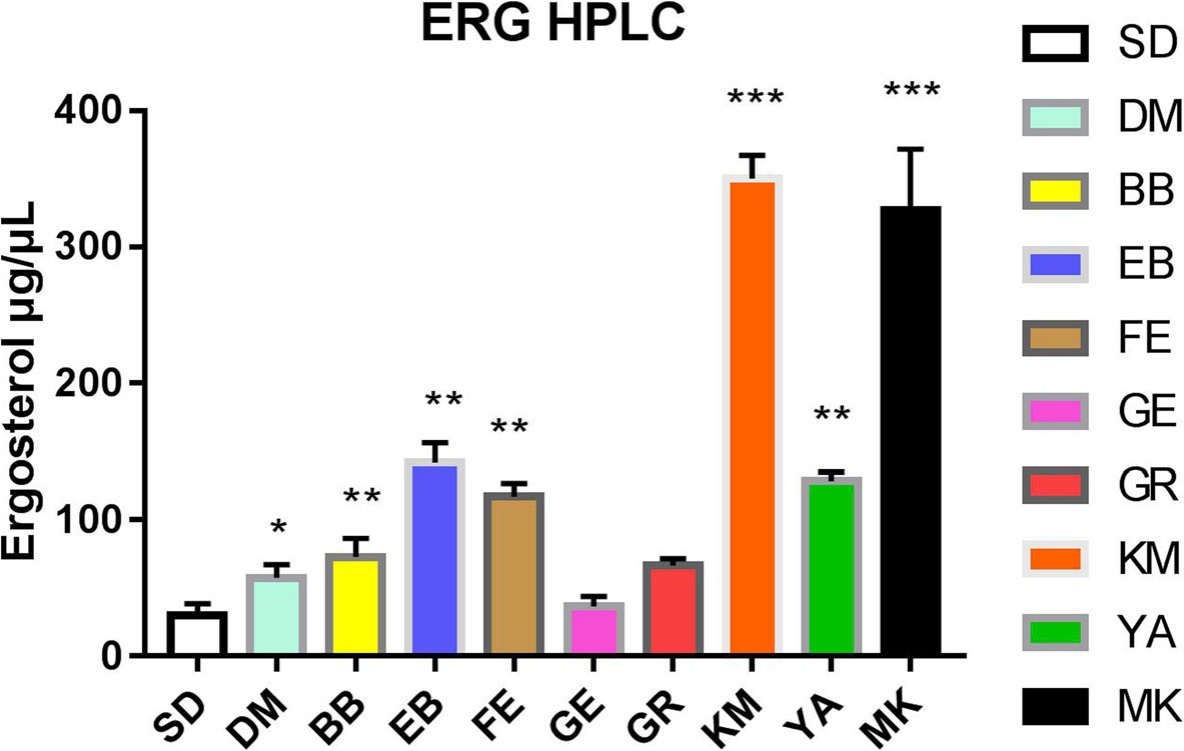
The ergosterol content of TM after treatment with different substances for 5 days was determined by HPLC and compared to the SDA control group. *P < 0.05; ** P < 0.01; ***P < 0.001. The following test substances were used: Sabouraud dextrose agar (SDA), Dimethyl sulfoxide (DMSO), berberine hydrochloride (BB), ethidium bromide (EB), Fenarimol (FE), genistein (GE), Congo red (GR), clotrimazole (KM), Adriamycin hydrochloride (YA), and Miconazole nitrate salt (MK)

Following treatment with different substances for 5 days, the ergosterol content of TM was determined by HPLC and compared to the SDA (control) group (Fig. 7). The ergosterol content of TM in the BB-, EB-, FE-, and YA-treated groups was significantly higher than that of the SDA (control) group (** *P* < 0.01). The ergosterol content of TM in the KM- and MK-treated groups was higher than that of the SDA (control) group with an increased significant difference (*** *P* < 0.001).

### The expression of cytochrome related gene of TM under KM, the difference of protein under KM and BB treatment, and the results of RT-PCR of CAMP related gene

**Fig. 8:**
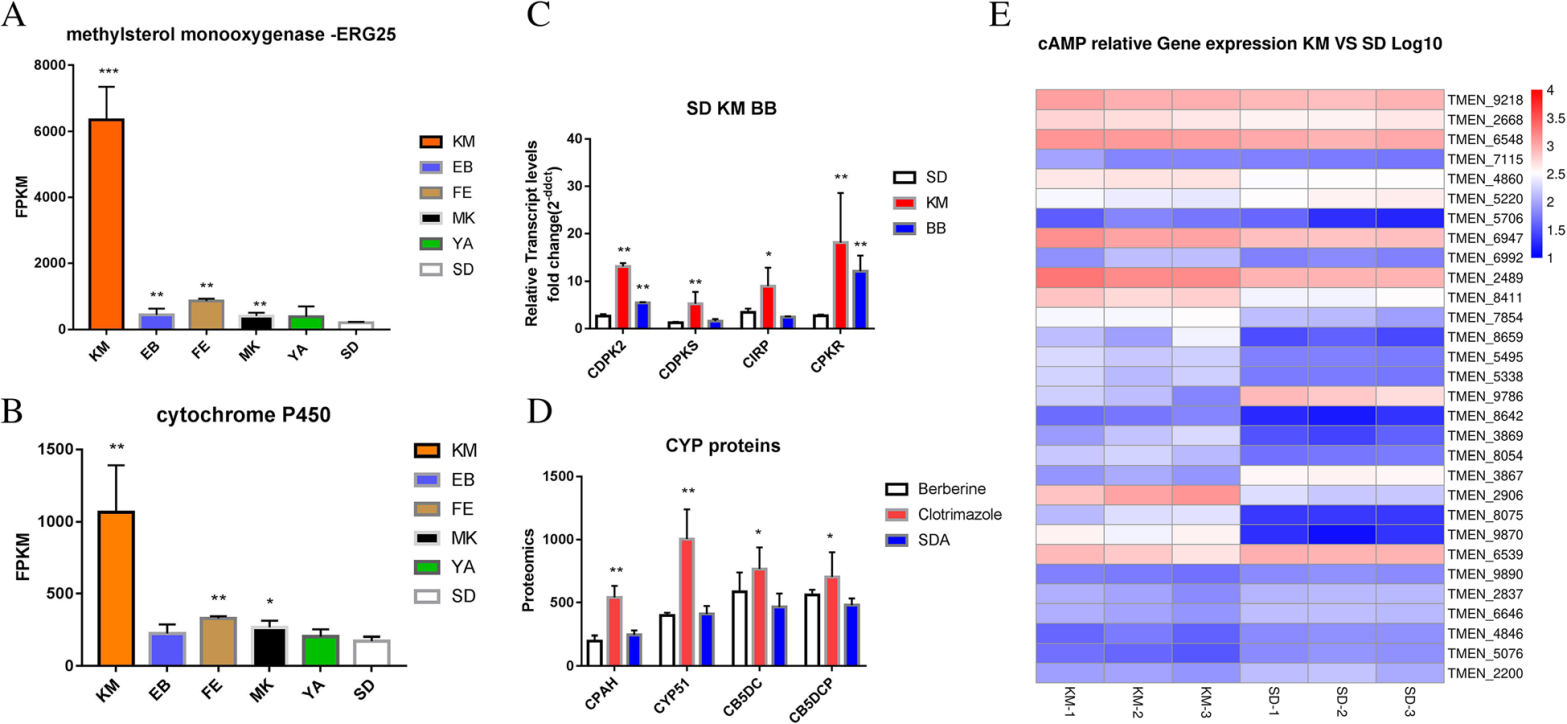
**a**: Transcriptome data analysis of methylsetol monooxygenase (ERG25) **b**: Transcriptome data analysis of Cytochrome P450 (CYP450) **c**:RT-PCR results of CPKR, CIRP, CDPK2 and CDPKS. **d**: Proteomic data of CYP proteins of TM treated with KM and BB **e**:Proteomic data of CAMP related proteins in TM treated with KM. Abbreviation means: CPKR means cAMP-dependent protein kinase regulatory subunit, CIRP means cAMP-independent regulatory protein, CDPK2 means cAMP-dependent protein kinase type 2, CDPKS means cAMP-dependent protein kinase catalytic subunit. CPAH means Cytochrome P450 alkane hydroxylase, CYP51 means Cytochrome P450 oxidoreductase, putative (CYP51), CB5DC means Cytochrome b5 heme-binding domain-containing protein, CB5DCP means Cytochrome b5 heme-binding domain-containing protein 2nd

Figure 8 shows the results of the transcriptome data analysis of methylsterol monooxygenase (ERG25) and cytochrome P450 (CYP450). A significant difference in the expression of the ERG25 gene and CYP450 gene (*P* < 0.05) was found in the KM-, EB-, FE-, MK-, and YA-treated groups,, which was most obvious in the KM- and FE- treated groups (*P* < 0.01). The relative gene expression of CAMP between the KM-treated group and the SDA control group is shown in Fig. 8e (Table 1). RT-PCR showed that the expression of cytochrome related genes including cAMP-dependent protein kinase regulatory subunit (CPKR), cAMP-independent regulatory protein (CIRP), cAMP-dependent protein kinase type 2 (CDPK2), and cAMP-dependent protein kinase catalytic subunit (CDPK) in the KM- and BB- treated groups were significantly higher than those in the SDA control group (*P* < 0.05).

**Table 1:** Description table of transcriptome Heatmap

Gene ID	Description
TMEN_9218	cytochrome c oxidase subunit 6A
TMEN_2668	cytochrome c oxidase polypeptide 5
TMEN_6548	cytochrome c oxidase subunit 6B
TMEN_7115	cytochrome c oxidase assembly protein
TMEN_4860	cytochrome P450 regulator
TMEN_5220	cytochrome c peroxidase
TMEN_5706	cytochrome P450
TMEN_6947	cytochrome c oxidase subunit 7A
TMEN_6992	cytochrome P450
TMEN_2489	cytochrome c oxidase subunit 4
TMEN_8411	cytochrome b5
TMEN_7854	cytochrome P450
TMEN_8659	cytochrome P450
TMEN_5495	NADPH--cytochrome P450 reductase
TMEN_5338	cytochrome P450
TMEN_9786	cytochrome P450
TMEN_8642	cytochrome P450
TMEN_3869	cytochrome P450
TMEN_8054	cytochrome b2
TMEN_3867	cytochrome P450
TMEN_2906	cytochrome P450
TMEN_8075	cytochrome P450
TMEN_9870	cytochrome P450
TMEN_6539	cytochrome c
TMEN_9890	cytochrome c oxidase assembly protein
TMEN_2837	cytochrome c oxidase assembly factor
TMEN_6646	cytochrome c oxidase assembly factor
TMEN_4846	NADH-cytochrome b5 reductase
TMEN_5076	cytochrome c1 heme lyase
TMEN_2200	cytochrome c oxidase-assembly factor

The results of proteomics in the KM-treated group showed that the CYP proteins including CYP450 alkane hydroxylase, CYP450 oxidoreductase, putative (CYP51), and cytochrome b5 heme-binding domain-containing protein were significantly increased compared to the SDA control group (P < 0.05), while no significant difference related to these proteins was found between the BB-treated group and the SDA control group (Table 2).

**Table 2:** Description of CYP relative proteins in proteomics experiment

	B			K			S		
Cytochrome P450 alkane hydroxylase	247.4	166.8	177.8	475.2	500.1	646.9	284.4	215.7	232.2
Cytochrome P450 oxidoreductase, putative (CYP51)	377.7	395.7	420.3	812.3	932.9	1266.7	479.8	391.6	355.7
Cytochrome b5 heme-binding domain-containing protein	480.1	515.6	762.2	769.7	936.4	595.7	580.1	444.2	367
Cytochrome b5 heme-binding domain-containing protein	515.3	596.2	571.4	928.3	609	575.6	425.4	533.5	478.8

### Biofilm thickness under KM and BB treatment

**Fig. 9:**
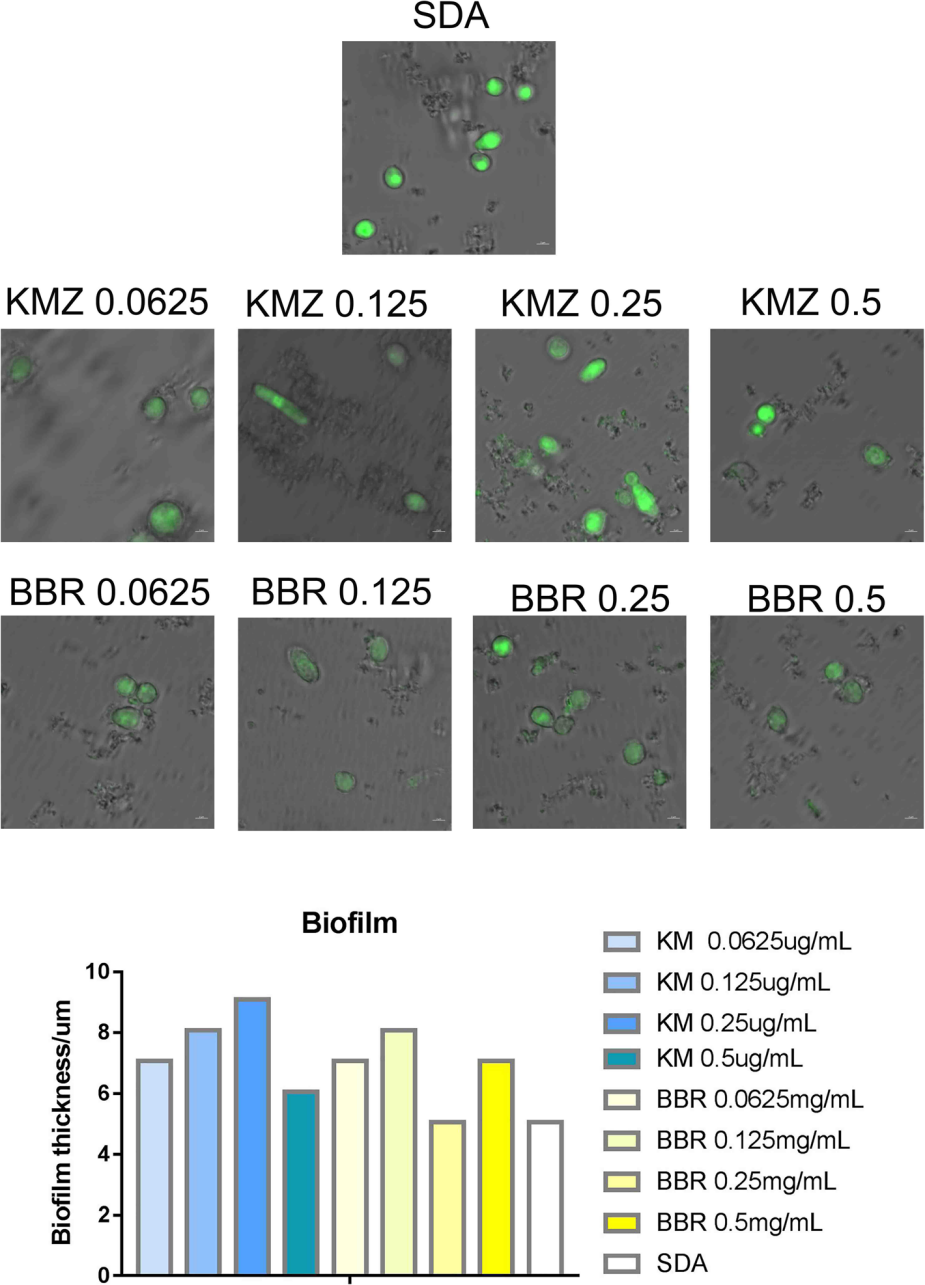
Biofilm thickness following exposure to specific concentrations of KM and BB

The results of the biofilm thickness of TM in the KM- and BB-treated groups showed that it gradually increased following exposure to specific concentrations of substances (Fig. 9). The biofilm thickness of TM gradually increased after increasing the concentration of KM from 0.0625 μg/mL to 0.25 μg/mL. The same result was observed when the concentration of BB was increased from 0.0625 mg/mL to 0.125 mg/mL.

## Discussion

Fungal infections have been increasing due to various factors, while present antifungal treatments show substantial complications due to their adverse effects and the rise of drug resistance. This continuous rise of fungal strains that are drug-resistant, represented by azole resistance, has become a serious threat to public health, [6]. At present, there are only a few studies on drug resistance of TM, and most studies only focus on functional analysis of some genes or localization of fluorescent proteins [7]. In this study, we selected several representative substances and used transcriptomics and proteomics research to analyze their mechanism of action on TM, and the corresponding strategies of TM in response to them. The results will lay a solid foundation for effective control and prevention of dermatomycosis due to TM in the future.

The experimental results of the influence of various substances on the growth curve of TM showed that its growth was obviously inhibited by higher concentrations of these substances. And with increasing concentrations, the antifungal effect also increased. In the subsequent electron microscope results, we observed some interesting phenomena. Some substances had obvious effects on the lipid production of TM at a concentration of 1 μg/mL. In particular, FE, EB, GR, KM, YA, and MK, and the results of ergosterol determination were consistent with the fluorescence value of lipids. This confirmed that these drugs have a significant effect on the lipid metabolism of TM. It seems that lipid metabolism is associated with fungal drug resistance. Ergosterol is the primary sterol, found in the plasma membranes of fungi, and plays an important role in membrane integrity and the activity of many enzymes bound to the membrane [8]. Inhibiting 14α-sterol demethylase results in a buildup of 14α-methylated sterols, which leads to a damaged cell membrane with less available ergosterol and modified permeability of the fungal cell [8]. Similar results have been found in Ma’s [9] research on the effect of Tebuconazole on *Fusarium graminearum*, in which the lipid expression gene of *Fusarium graminearum* was significantly up-regulated. The transcriptome and fluorescence quantitative PCR results of this study showed that the expression of the ERG25 gene, related to fat metabolism, was significantly up-regulated following exposure to KM, EB, FE, MK, and YA, with KM showing the most prominent up-regulation. ERG25 catalyzes oxidation of the ergosterol intermediate 4,4-dimethylzymosterol at the C-4 position, is comprised of three histidine-rich clusters, which are common in iron-binding non-heme enzymes, contains an endoplasmic-reticulum retrieval signal, KKXX [10], and is part of the oxidoreductase family [11]. The genes that encode ERG25 have been cloned from the fungus *Penicillium chrysogenum* (*P. chrysogenum*), which can produce penicillin. During production of penicillin, the intermediates and enzymes of the penicillin biosynthetic pathway have to be conveyed across multiple membranes. Then, the produced hydrophobic penicillin needs to be delivered into the medium through the peroxisome as well as the plasma membranes. Therefore, transport activities are important in the biosynthesis of penicillin. Ergosterol plays a major part in the permeability of penicillin through the membrane of *P. chrysogenum* [11]. Previous research indicated that ERG6 was highly up-regulated after treatment with azole fungicides, which had a 37.5 and 50.3 fold increase in expression in Trichophyton rubrum and *C. albicans*, respectively [9]. The results of this study have shown that the expression of ERG25 was significantly up-regulated, especially after treatment with KM, EB, FE, and MK, and indicated that the liposome production of TM was significantly enhanced by KM, FE, and MK. These results made us wonder; why does the liposome content of TM only significantly change under the influence of certain substances? There may be three possible reasons for this. Firstly these substances may promote the upstream genes of ERG in fungi, resulting in their up-regulation. Secondly, liposomes protect fungi to a certain degree. Since the permeability of *P. chrysogenum* is related to the ERG gene, KM, FE, and MK could also induce the permeability of TM’s cell membrane. By up-regulating the expression of ERG25 and other genes, the content of liposomes in the cell membrane increases, thereby increasing the permeability of these substances, resulting in a reduced accumulation of them, which is a self-protective mechanism of fungi. Finally, the third reason may be that the metabolites of liposomes could neutralize certain chemical substances and fungi can protect themselves by enhancing the metabolism level of liposomes.

In a previous study by Ma et al. was discovered that the expression of 18 genes that encode components of the cAMP had significantly increased after the fungus was treated with KM [12], which is consistent with previous findings that the ergosterol biosynthetic pathway is targeted by azole fungicides and reacts to changes in ergosterol levels. According to previous research, fungal CYP450 may play an important part in the detoxification of fungicides or their metabolites [12]. Therefore, it seemed interesting to evaluate if the expression of ERG genes in TM are regulated by this transcription factor. In our study, we found that the CYP450 gene and other related genes were significantly up-regulated by MK, FE, and especially KM, indicating that it had a regulatory role on cAMP related genes. It has been observed previously that after treating *F. graminearum* with tebuconazole, the two genes, FGSG_03498 and FGSG_09195, which encode the CYP450 monooxygenase, were up-regulated by 25.39 and 149.95 folds, respectively [9]. Similar results of the reaction of fungi to fungicides have been frequently observed [12, 13], indicating the possibility that fungal CYP450 may play an key part in detoxifying fungicides or their metabolites [14].

CYP450s are part of a large hemease superfamily. They are the first set of enzymes labelled as “superfamily,” since CYP450s have important roles to play in both the primary and secondary metabolic pathways [15]. These enzymes are important in the catalyzation of oxidative processes of multiple organic substrates and have a key role in steroid conversion and heterogeneous metabolism in the biological domain [6]. Bioactivation of drugs, mediated by CYPs, may cause oxidative stress and result in pathophysiology. The use of drugs may induce hypersensitivity reactions, whereas a metabolic inclination to drug hypersensitivity worsens it. Primarily, various intermediate bioactive products of drug metabolism, mediated by CYPs, are the main cause. Therefore, why do these substances have an up-regulation effect on TM, while the CYP gene itself is part of the regulation of the steroid biosynthesis pathway? Since the regulation of the CYP gene is closely related to fat metabolism and synthesis, CYP related genes will be up-regulated after drug treatment. In a previous study, the impact of the induction of steroid on the resistance of antibiotics targeting the fungal steroid fusidic acid, ramycin; 16-(acetyloxy)-3α,11α-dihydroxy-29-dammara-17(20),24-dien-21-oic-acid, as well as on the reduction of carbonyl, and degeneration of the novel anti-insect agent NKI 42255 (2-(1-imidazolyl)-1-(4-methoxyphenyl)-2-methyl-1-propanone) were investigated in Comamonas testosterone, which is a Gram-negative soil bacterium [16]. The results clearly showed that the induction of steroid made it possible for the bacterium to exist in a milieu that would otherwise be deadly if it was uninduced. This indicates that TM up-regulates the steroid biosynthesis gene under the action of certain substances, which benefits the self-survival of fungi and therefore TM, in harsh environments by producing excessive steroid.

Biofilm, the tool for survival in fungal colony formation, is a type of three-dimensional structure produced by the microbial community for self-protection. It can adhere to the surface of tissue and is wrapped by extracellular polymer matrix (ECM), which is produced by itself. The formation of biofilm is affected by many factors, including the host’s immune state, nutrients, drugs, and physical and chemical factors of the growth environment. Biofilm can lead to fungal drug resistance, and is related to the ERG and CYP [17] genes. The results of a previous study indicate that treatment with fluconazole could affect the metabolism of the fungal structure and cell wall [18, 19], and that these effects could be steadily integrated into the cell wall, after the addition of resistance [20]. Recently, researchers have examined the cAMP signaling pathway in relation to modifying the sensitivity towards antifungal azoles. Mutants of *C. albicans*, in the genes that encode the proteins in charge of cAMP synthesis, have shown great hypersensitivity to fluconazole and also to other inhibitors of sterol biosynthesis [21]. Adding cAMP resulted in partial-to-complete reversion of this hypersensitivity. These results indicate that adenylate cyclase inhibitors are capable of modifying antifungal susceptibility.

We found, through transcriptome data and RT-PCR, that the values of elongation factor 3-ELFT (EF3), were significantly up-regulated by BB, MK, FE, EB, and YA. EF3 has a crucial role in the translation of protein [22] and regulates the binding affinities of the deacylated tRNA and the ternary complex to the ribosomal E and A site, respectively. This way, EF3 seems to keep a balance between the amino acid fidelity and protein translation rate. Since EF3 is a vital protein, repression of the antisense controlled by an inducible promoter, has presented a suitable way to modify the function of the EF3 gene [23]. The existence of HEAT repeats, attribute to increased evidence of the potential role of EF3 in targeting protein, and serve as protein-protein interaction domains [22]. Another study showed that a slow growing phenotype of EF3, found in *P. infestans*, is possibly related to defects in the synthesis of protein. Their results have suggested that with overexpression of EF3 in *P. infestans*, a small elongation defect still exists, which seems to be consistent with the slight sensitivity to antibiotics found in this strain [24]. The up-regulation of EF3 indicates that fungi can improve the synthesis of transport proteins under the stress of drug effects, in order to cope with the threat of drug stimulation.

## Conclusions

In this study, the biofilm of TM was enhanced under the stimulation of certain substances, which is related to the up-regulation of proteins related to the ERG, CYP, and EF3 genes. The results suggests that it is necessary to inhibit the expression of ERG, CYP, and EF3 related genes as well as fat metabolism in order to effectively deal with dermatomycosis caused by TM. These inhibitions lead in turn to the inhibition of biofilm production by the fungus, which can potentially solve the problem of fungal drug resistance in clinical settings.

## Methods

### Test strains

*Trichophyton mentagrophytes* strains were isolated from rabbits showing symptoms of dermatophytosis, which were provided by the Institute of Internal Medicine, Shaoxing District. The presence of the TM strains was confirmed by The Chinese Academy of Medical Sciences and subsequently grown in SDA at 28 °C with 60% moisture. TM were grown in Sabouraud’s Glucose Agar Medium (SDA) at 28 °C with 60% moisture for 14 days before the experiment.

### Growth curve by OD value

The following substances were used in the experiment: BB (Lot No. 20130306) was produced by Shanghai Yuanye Bio-Technology Co., Ltd., China. KM (Lot No. 23593–75-1) was obtained from BaDaTong Medical Company, China. GE (Batch: 02) was purchased from Extrasynthesis Genay Cedex, France. FE (Lot No. C13430000) was purchased from Dr. Ehrenstorfer GmbH Co., Ltd., Germany. GR (Lot No. M180920979), EB (Lot No. C10111224), YA (Lot No. C10223862), and MK (Lot No. C10038681) were purchased from Shanghai Macklin Biochemical Co., Ltd., China. DM (Lot No. 0231) was purchased from Amresco, USA. SDA (Lot No. 403Q021) was purchased from Solarbio Life Sciences, China, whose purity was not lower than 98%.

Concentration and time effects of different substances on TM were established as previously proposed. TM were grown in SDA at 28 °C with 60% moisture for 14 days before the experiment. The spores were washed down through the Sabouraud glucose liquid medium (SDB). The washed liquid was filtered through double-layer filter paper. The density of the suspension determined by a blood cell counter. Mycelium as a homogenate. In short, the fungi were cultured, in presence of BB (0.01, 0.1, or 1 μg /mL), in a concentration of 30,000 cells/mL [25] at 37 °C for 0, 2, 4, 6, 8, 10, 12, 14, 16, 18, 20, 22, 24, 26, 28, 30, 32, 34, 36, 38, 40, 42, 44, 46, 48, 50, 52, 54, 56, 58, and 60 h. The growth of TM was observed through a Automatic growth curve analyzer (Bioscreen C, FP-1100-C, Helsinki, Finland) with an OD value of 540 nm [26].

### Ultra-structural analysis by electron microscopy

TM were grown in SDA at 28 °C with 60% moisture for 14 days. All the substances were dissolved in DMSO and prepared with a concentration of 1 μg/mL. With a cork bore the fungus was cut into round blocks and inoculated on the drug sensitive include BB, KM, GE, FE, GR,EB, YA, MK and DM plate to incubate for 10 days.

For TEM, the specimens were first fixated with 2.5% glutaraldehyde in phosphate buffer (0.1 M, pH 7.0; > 4 h) and then post-fixated with 1% OsO4 in phosphate buffer for 1-2 h. Following washing, graded ethanol was used to dehydrate the specimens and acetone was used to incubate them. After embedding with resin, the specimens were cut into ultra-thin sections on a LEICA EM UC7 ultratome, followed by staining with alkaline lead citrate and uranyl acetate (5–10 min each) and finished with an analysis under a Hitachi Model H-7650 TEM.

For SEM analysis, the specimens were prepared as outlined above for TEM. Then, they were placed for 30 min in ethanol:isoamyl acetate (1:1) and then isoamyl acetate during the night. After dehydrating the specimens in a Hitachi Model HCP-2 critical point dryer containing liquid CO_2_, they were covered by gold-palladium with a Hitachi Model E-1010 ion sputter (for 4–5 min) and analyzed with a Hitachi Model TM-1000 SEM.

### Nile red staining and ergosterol content determination

After 5 days of treatment with test substances, the strains were placed on a clean glass plate with a toothpick, stained with Nile red at a concentration of 50 μg/ml with 20 μl added, and gently smeared. The staining was observed under a Zeiss confocal microscope and the fluorescence was analyzed by Image J software. The content of ergosterol was determined by high performance liquid chromatography (HPLC). First, 0.1000 g of sample powder was accurately weighed and grounded by liquid nitrogen, then added to a 2 ml volumetric flask with 7 m acetonitrile added to it. Next, the mixture was thoroughly mixed, placed into a water bath at 50 °C, taken out after 40 min for an ultrasonic extraction at room temperature for 30 min, then cooled to room temperature, and fixed with acetonitrile. For testing, the mixture was mixed well, then 1 mL of supernatant was taken and passed through a 0.22 μm filter membrane. Test conditions were set as follows: detector: DAD, C18 column: 250*4.6 mm; 0.5 μm, column temperature: 25 °C, wavelength: 281 nm, mobile phase: methanol: water = 80:20 (V:V), flow rate: 1 ml/min, injection volume: 10 μL.

### Biofilm thickness under KM and BB treatment

The spore suspension of TM (5 × 10^7^ CFU/ml) at a logarithmic growth stage was diluted with DMEM basic medium and statically cultured for 6 h on a round coverslip (BS-14-RC) (Biosharp, Life sciences, Anhui, China). Then, BB and KMZ medium was added in the following concentrations for 16 h of static culture: 0 .0625 mg/mL, 0.125 mg/mL, 0.25 mg/mL, and 0.5 mg/mL. After dark staining with the Fun1 cell stain (10 μM, Invitrogen, USA) for 1 h, the biofilm of the strain was observed with a laser confocal microscope (Zeiss, LSM880, Germany) at the excitation wavelength of 405 nm and 410–480 nm. The fluorescent three-dimensional structure map was constructed with Image J software.

### mRNA library construction and sequencing

All the test substances including BB, KM, GE, FE, GR,EB, YA, MK and DM were dissolved in DMSO and prepared at a concentration of 1 μg/mL. Three biological replicates were prepared for each condition.

TRIzol Reagent (Invitrogen) was used for extraction of total RNA, per manufacturer’s instructions [25]. The total RNA quantity and purity were analysis of Bioanalyzer 2100 and RNA 6000 Nano LabChip Kit (Agilent, CA, USA) with RIN number > 7.0.Approximately 10 μg of total RNArepresenting a specific adipose type was subjected to isolate Poly (A) mRNA with poly-T oligoattached magnetic beads (Invitrogen) [27]. Following purification, the mRNA is fragmented into smallpieces using divalent cations under elevated temperature. Then the cleaved RNA fragments werereverse-transcribed to create the final cDNA library in accordance with the protocol for the mRNASeqsample preparation kit (Illumina, San Diego, USA), the average insert size for the paired-endlibraries was 300 bp (±50 bp). And then we performed the paired-end sequencing on an IlluminaHiseq4000 at the (LC Sceiences,USA) following the vendor’s recommended protocol.

#### Sequence and primary analysis

A cDNA library constructed by technology from the pooled RNA was sequenced run with Illumina 4000 sequence platform. Using the Illumina paired-end RNA-seq approach, we sequenced the transcriptome, generating a total of millon paired-end reads of bp length. This yielded gigabases (Gb) of sequence. Prior to assembly, the low quality reads (1,reads containing sequencing adaptors; 2,reads containing sequencing primer;3, nucleotide with q quality score lower than 20) were removed. After that, a total of G bp of cleaned,paired-end reads were produced. The raw sequence data have been submitted to the NCBI Short Read Archive with accession number.

#### RNA-seq reads mapping

We aligned reads of sample A and sample B to the UCSC (http://genome.ucsc.edu/) *Homo sapiens* reference genome using HISAT package, which initially remove a portion of the reads based on quality information accompanying each read and then maps the reads to the reference genome. HISAT allows multiple alignments pe read (up to 20 by default) and a maximum of two mismatchs when mapping the reads to the reference. HISAT build a database of potential splice junctions and confirms these by comparing the previously unmapped reads against the database of putative junctions.

#### Transcript abundance estimation and differentially expressed testing

The mapped reads of each sample were assembled using StringTie. Then, all transcriptomes from Samples were merged to reconstruct a comprehensive transcriptome using perl scripts. After the final transcriptome was generated, StringTie and edgeR was used to estimate the expression levels of all transcripts. StringTie was used to perform expression level for mRNAs by calculating FPKM. The differentially expressed mRNAs and genes were selected with log2 (fold change) > 1 or log2 (fold change) < − 1 and with statistical significance (*p* value < 0.05) by R package.

### Real-time PCR

With a Promega kit (Madison, WI, US), first cDNA was acquired from total RNA (1 μg) per manufacturer’s instructions. Quantitative RT-PCR was conducted with an ABI StepOnePlus (Applied Biosystems) paired with SYBR Green Supermix (TaKaRa) per manufacturer’s instructions. Amplification was conducted at 94 °C for 10 m, then 40 cycles at 94 °C for 15 s, 60 °C for 31 s, and 72 °C for 1 m. Table 3 contains a description of the primers. Cycle threshold (CT) values were acquired and the 2 − ΔΔCt method was used to analyze the data [28]. 18S, an endogenous housekeeping gene, was used to carry out normalization. The relative expression levels of mRNA from triplicate experiments were expressed as mean ± SD.

**Table 3:** Primer sequences for RT-PCR validation

Name	Sequence	Product length
AFTA-up	CAAGTCGAATGAAGCGAGCG	78 bp
AFTA-dn	AGTGACCGAACTGTGCGAAT	
ELFT-up	AGACCGCCATCAAGCGTAAA	92 bp
ELFT-dn	CTTTGGCAAGAAAGCAGCGA	
STPK-up	ATGTAAGGCAAGGAACCGCA	114 bp
STPK-dn	AGAGGTCTTCTCGGGTCTCC	
18S-up	AGACCTGGAACCTTTGACGC	73 bp
18S-dn	CATCGCTTGTCTCGGCATTG	
CPKR-up	CAGCTACCGTCATCTCGACC	166 bp
CPKR-dn	ATCTTGGAGCGCTCATACGG	
CIRP-up	CTCCATGACTTGCCGTCTGT	189 bp
CIRP-dn	ATATCACGGCCATGTTCGCA	
CDPK2-up	CTGGACTGGGCTGTATGACG	129 bp
CDPK2-dn	GCGGACGAAGGACTCGAAAT	
CDPKS-up	GGAGCAAGGTATTCCGGTGT	146 bp
CDPKS-dn	CATGGAATAGCCTACCGCGA	

### Statistical analysis

The Duncan’s test was performed to compare groups. Statistical analyses were conducted with GraphPad Prism 5 and *P* < 0.05 was considered as statistically significant. A Pearson correlation was used to statistically analyze the agreement between qRT-PCR analysis and Illumina RNA-seq analysis.

### Protein extraction and trypsin digestion

BB and KM were dissolved in DMSO and prepared at a concentration of 1 μg/mL. Trypsin digestion and protein extraction were conducted as described previously [29]. The specimens were first grounded in tubes of 5 mL and then treated in an ultrasonic processor of high intensity. The solid material obtained through centrifugation at 4 °C was disposed. The material in the supernatant was first precipitated in 15% trichloroacetic acid for 2 h and then placed in pre-cooled acetone. Then, the material that was recovered, was ground first in ammonium bicarbonate buffer, then recovered, and lastly alkylated. Following digestion with trypsin gold (Promega, Madison, WI, USA) multiple types of peptides were produced. To achieve digestion on the first day, we first added trypsin at the highest ratio of 1:50, then at a ratio of 1:100 for the following 4 h of digestion. About 100 μg of every protein sample was required for TMT labeling.

### TMT labeling, high performance liquid chromatography fractionation, and LC-MS/ MS analysis

We extracted salt from the specimens, labeled it with a Strata X C18 column (Phenomenex, Torrance, CA, USA), and vacuum-dried it through centrifugation. To perform TMT labeling as described previously [29], a 6-plex TMT Kit (Thermo Fisher Scientific, Waltham, MA, USA) was used to reconstitute in 0.5-M triethylammonium bicarbonate buffer. To mark 100 μg of the target specimen, one section of TMT reagent was used. Then, the mixture of specimen and reagent was left at room temperature for 2 h. Next, the salt was eliminated and the mixture dehydrated. A model 300 Extend C18 column (5 μm particles, 4.6 mm ID, 250 mm length, pH 10; Agilent, Santa Clara, CA, USA) was used to partition the specimens based on a 2 to 60% gradient of acetonitrile supplemented with 10 mM of ammonium bicarbonate for 80 min. The resolved fractions were combined and water was eliminated. LC-MS/MS analysis was performed at the Jingjie PTM BioLab (Hangzhou) Co., Ltd. (Hangzhou,China) as described previously [29].

### Database search

Thermo Proteome Discovererver. 2.1.0.81 software and the Mascot search engine were used to analyze the MS/MS data. The unip_Anas_8839 database, retrieved from Uniprot (https://www.uniprot.org/ proteomes/?query = taxonomy:8839), was included in the search., Trypsin/P was used as the cleavage enzyme. The enzyme only allowed two missing cleavages, five alterations, and five charges per peptide. In accordance to previous studies, faults in quality allowance were predetermined as 0.02 Da for fragment ions and 10 ppm for precursor ions [30, 31]. TMT-6-plex on lysine and carbamido-methylation on cysteine was regarded as stable, and acetylation on protein N-term and oxidation on methionine as changeable elements (Tables S1 and S2). The FDRs of peptides and sites of modification were only allowed an error value of 0.01. The peptide length was set at a minimum of 7. The default parameters were predetermined in Thermo Proteome Discoverer. The unique peptide quantities are summarized in Tables S1 and S2. We used raw materials to calculate the intensities of the TMT reporter ion by MS/MS as well as the fold changes of the specimens to perform quantification of TMT. To centralize the quantitative distribution of each specimen, they were mean normalized in the middle range. Then, the protein quantitation was calculated, which served as the median ratio of the corresponding razor or unique peptides of a specific protein. To compare the proteins’ accumulation, two-sample and two-sided tests were used. Generally, in statistical analysis, a *P* value of < 0.05 was considered and reported as significant. Data on MS proteomics was deposited in the ProteomeXchange Consortium via the PRIDE partner repository (http://proteomecentral.proteomexchange.org/) with the data set identifier GEO Submission (GSE162331) [NCBI tracking system #21491828].

### Bioinformatic analysis

GO data was obtained from the UniProt-GOA database (http://www.ebi.ac.uk/GOA/). Although the protein IDs s were adapted to the UniProt IDs, they could simultaneously be labelled to the GO maps. The method of protein sequence alignment was used to note the unannotated proteins. Furthermore, the KEGG database (http://www.genome.jp/kegg/) was also reviewed to detect biological and metabolic reactions. KAAS, a KEGG online service tool, was used to mark every protein’s KEGG description. Then, the KEGG pathway from another KEGG service product was adapted to map the proteins. To predict the subcellular localizations in each recognized protein, a new version of PSORT/PSORT II (http://www.genscript.com/wolf-psort.html) was used. InterProScan sequence analysis software including the protein sequence alignment method (http://www.ebi.ac.uk/interpro/) was adapted for various analyses of proteins such as its domain.

## Supplementary Information

**Additional file 1: Table S1.** Information of Proteins Unique Sequence ID, Accession, Description and Sequence. (XLS 2984 kb)

**Additional file 2: Table S2.** Information of Peptide Groups Peptide Group ID, Annotated Sequence, Qvality PEP. (XLS 1255 kb)

## Data Availability

The raw transcriptome data has been deposited in the Sequence Read Archive of the National Center for Biotechnology Information (NCBI) (accession No. GSE162331 and [NCBI tracking system #21491828]). The datasets used and/or analysed during the current study are also available from the corresponding author on reasonable request.
