# Correction to: Assessment of the mechanism of drug resistance in *Trichophyton mentagrophytes* in response to various substances

**DOI:** 10.1186/s12864-021-07630-1

**Published:** 2021-05-19

**Authors:** Chenwen Xiao, Jiaoyu Wang, Zhenfeng Liao, Yee Huang, Quanan Ji, Yan Liu, Fei Su, Lijun Xu, Qiang Wei, Yao Pan, Ke Li, Guolian Bao

**Affiliations:** 1 Institute of Animal Husbandry and Veterinary Science, Zhejiang Academy of Agricultural Sciences, Hangzhou, China; 2 State Key Laboratory for Managing Biotic and Chemical Treats to the Quality and Safety of Agro-Products, Institute of Plant Protection and Microbiology, Zhejiang Academy of Agricultural Sciences, Hangzhou, 310021 China; 3 Central Laboratory of Zhejiang Academy of Agricultural Sciences, Zhejiang Academy of Agricultural Sciences, Hangzhou, China; 4 National Clinical Research Center for Infectious Diseases, The Department of Infectious diseases, State Key Laboratory for Diagnosis and Treatment of Infectious Diseases, The First Affiliated Hospital, College of Medicine, Zhejiang University, 79 Qingchun Road, Hangzhou, 310003 China; 5 College of Life Sciences, China Metrology University, Hangzhou, China

**Correction to: BMC Genomics 22, 250 (2021)**

**https://doi.org/10.1186/s12864-021-07520-6**

Following publication of the original article [1], it was reported that there was an error in Fig. 3. The labels for each panel were missing in the original publication. The correct Fig. 3 is given in this Correction article and the original article has been corrected.

**Fig. 3:**
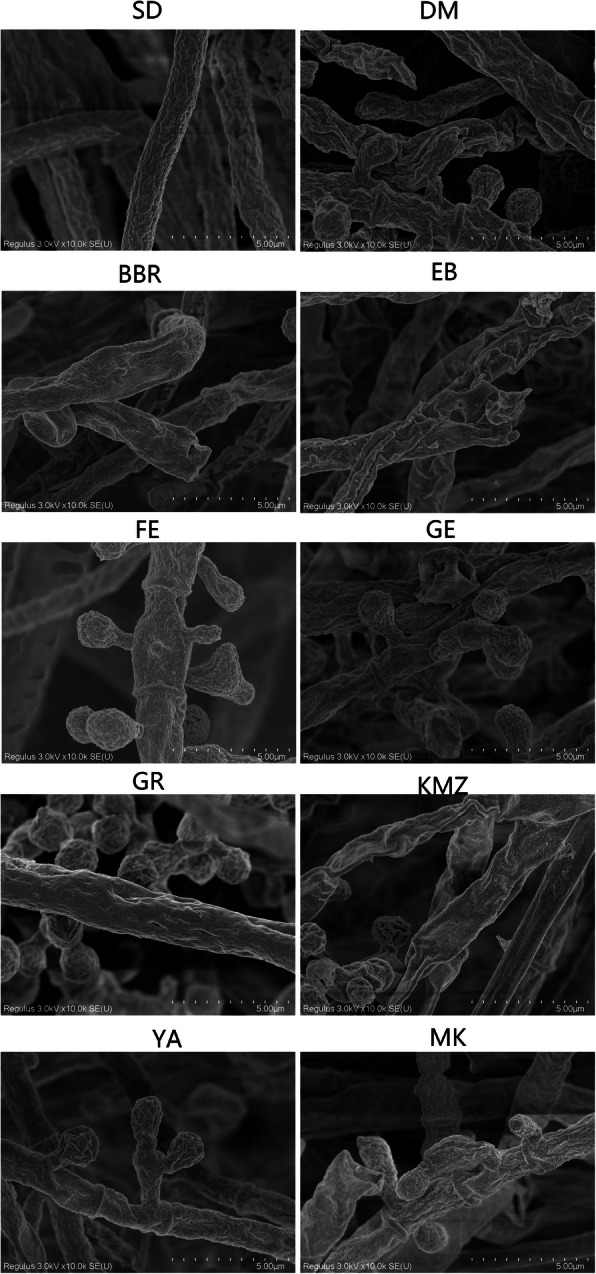
SEM analysis of the TM specimens following exposure to different drugs substances. The specimens were placed in ethanol:iso-amyl acetate (1:1) for 30 min, and then in iso-amyl acetate overnight
